# Urolithiasis Develops Endothelial Dysfunction as a Clinical Feature

**DOI:** 10.3390/antiox10050722

**Published:** 2021-05-04

**Authors:** Javier Sáenz-Medina, María Martinez, Silvia Rosado, Manuel Durán, Dolores Prieto, Joaquín Carballido

**Affiliations:** 1 Department of Urology, Puerta de Hierro-Majadahonda University Hospital, 28222 Majadahonda, Spain; 2 Department of Medical Specialities and Public Health, Faculty of Health Sciences, King Juan Carlos University, 28933 Móstoles, Spain; manuel.duran@hospitalreyjuancarlos.es; 3 Department of Nephrology, Clinic University Hospital Valladolid, 47003 Valladolid, Spain; mariamartinezmanrique@gmail.com; 4 Biobank, Health Research Institute “Puerta de Hierro Segovia de Arana”, 28222 Madrid, Spain; srosado@idiphim.org; 5 Department of Physiology, Pharmacy Faculty, Complutense University, 28040 Madrid, Spain; dprieto@ucm.es; 6 Department of Urology, Puerta de Hierro-Majadahonda University Hospital, Autonoma University, 08193 Bellaterra, Spain; joaquinalberto.carballido@salud.madrid.org

**Keywords:** endothelial dysfunction, oxidative stress, urolithiasis

## Abstract

An increased risk of cardiovascular morbidity has been reported in lithiasic patients. In this context, endothelial dysfunction (ED), an earlier status of atherogenesis, has been identified in hyperoxaluria rat models of urolithiasis. Objective: The purpose of this study was to determine the endothelial vascular function in patients with urolithiasis in relation to systemic inflammatory, oxidative stress, and vascular function serum markers. Methods: A cross-sectional study was performed between 27 urolithiasic patients, matched for age and sex, with 27 healthy patients. Endothelial function was assessed by measuring flow-mediated dilation (Celermajer method). Fasting blood was collected to determine metabolic parameters (glucose and lipid profile), along with serum CRP, IL-6, MDA, ADMA, and VCAM-1. Results: Both the control and urolithiasis groups were homogenous in anthropometric, exploration, and general laboratory measures. Flow-mediated dilation (%FMD) was 11.85% (SE: 2.78) lower in the lithiasis group (*p* < 0.001). No significant differences were achieved between groups when CRP, IL-6, MDA, ADMA, and VCAM-1 were compared, although slightly higher values of CRP, ADMA, and VCAM-1 were detected in the lithiasic group. A correlation was not reached in any of the serum markers when they were related to flow-mediated values, although a slight negative correlation trend was observed in MDA, VCAM-1, and IL-6 values. Conclusions: Endothelial dysfunction constitutes an important disorder related to urolithiasis patients. It must be considered as an early feature responsible for future cardiovascular events. Our study did not find a significant association between inflammatory, oxidative stress, endothelial serum markers, and flow-mediated dilation.

## 1. Introduction

Nephrolithiasis is a worldwide public health problem affecting between 5% and 9% of the European population and almost 12% of the North American society nowadays [1].

Urolithiasis by itself has been associated to an increased risk of cardiovascular diseases. An increase of myocardial infarction risk up to 31% was reported in a large longitudinal study for patients with a history of renal stones [2]. On the other hand, in a study of over 200 patients Aydin et al. reported a relationship between urinary calcium, oxalate excretion, and Framingham and SCORE risk scores, thus determining that lithiasic patients carry a high risk of cardiovascular disease and mortality [3].

Endothelial dysfunction (ED) is a well-established disorder consisting of impaired vasodilation, angiogenesis, and barrier function, along with an elevated expression of pro-inflammatory and pro-thrombotic factors in which oxidative stress (OS) is implied [4]. It is considered a key element in the development of atherosclerosis cardiovascular disease and has been associated with systemic inflammatory disorders such as diabetes, obesity, or metabolic syndrome. Hyperoxaluria has also been reported to cause systemic ED both in animal models and cell culture studies [5,6].

Augmented ROS production activates the oxidative stress sensitive nuclear transcription factor (NF-kB) that directly upregulates NADPH oxidase and regulates the expression of genes encoding adhesion molecules, COX-2, and pro-inflammatory cytokines TNFα, IL-6, and C-reactive protein (CRP), which in turn may activate NADPH oxidase and ROS generation, thus impairing endothelial function [4].

Previous studies in human hypertension diabetes mellitus (DM) or obesity have reported that molecular processes underlying vascular injury involve reactive oxygen species (ROS) and the activation of redox-sensitive signaling pathways.

ROS are also implicated in the pathogenic mechanisms of stone formation through endothelial injury, as an important step that triggers the growth of the stone over Randall’s plaque in the renal papilla or over the Bellini duct [7].

Oxidative stress is a common pathogenic factor for cardiovascular morbidities and for the development of nephrolithiasis, and no studies assessed demographics and stone characteristics along with stress markers in relation with ED. The objective of this study was to evaluate whether patients with urolithiasis exhibit systemic endothelial dysfunction in comparison with healthy patients through the Celermajer method, along with stone characteristics, serum markers of oxidative stress (MDA), endothelial dysfunction (ADMA, VCAM-1), and inflammation (IL-6 and CRP) in a cross-sectional study of lithiasic and healthy patients.

## 2. Material and Methods

### 2.1. Study Design

A cross-sectional study was performed between 27 urolithiasic patients, matched for age and sex, and 27 healthy patients. All of them were recruited from the outpatient clinic of the Urology Department of Puerta de Hierro University Hospital.

Every patient was evaluated through a clinical history and physical examination. Clinical data, previous pathologies, and blood analysis were collected to exclude patients with metabolic, respiratory, cardiovascular, hepatic, or renal disorders. Patients in treatment with drugs, which could affect the endothelial function, were also discarded. Abdominal echography, as a routinary part of the checkup, demonstrated the absence of urolithiasis in the control group. 

The ethical committee of Puerta de Hierro University Hospital, according to the Spanish legislation LOPD 15/1999 and its adaptation to the European Union RD 5/2018, approved the study. All the individuals signed informed consent to access to the study.

### 2.2. Assessment of Endothelial Function

After the urological and physical evaluation, endothelial function was evaluated using the Celermajer method by a blinded vascular radiologist [8] with high-resolution Doppler ultrasonography. Measurement of the brachial artery diameter was carried out before and after 5-min ischemic compression with a blood pressure cuff inflated around the forearm. Measurement of maximal post ischemic compression artery diameter was taken one minute after cuff release and related with precompression data. The difference between both lumen diameters expressed as the percentage change was considered as the endothelium-dependent vasodilatation (FMD increase %).

The Framingham risk-scoring table was used for the assessment of developing coronary heart disease, in a 10-year risk of developing coronary heart disease. The risk factors introduced into the calculator (mdcalc.com) were as follows: age, sex, smoker, total cholesterol, HDL cholesterol, and systolic blood pressure.

The atherogenic index was calculated as a logarithmic transformation of the ratio of triglycerides to HDL cholesterol [9].

### 2.3. Assays

Samples from patients included in this study were provided by the biobank of the health research institute “Puerta de Hierro Segovia de Arana” (PT17/0015/0020 in the Spanish National Biobanks Network), they were processed following standard operating procedures with the appropriate approval of the Ethics and Scientific Committees”.

In total, 5 mL of fasting blood was collected with EDTA and used for the determination of glucose, total cholesterol, HDL and LDL cholesterol, triglycerides, and C-reactive protein (CRP) using commercially available kits. Serum levels of interleukin-6 (IL-6), malondialdehyde (MDA), vascular cell adhesion protein 1 (VCAM-1), and asymmetric dimethylarginine (ADMA) were estimated using commercially available ELISA kits (VCAM-1 and ADMA: Elabscience Biotech, IL-6: Cayman Chemical, MDA MyBioSource).

### 2.4. Data Presentation and Statistical Analysis

Statistical analysis was performed using Excel, SPSS, and Prisma software packages. Differences between groups were assessed by Student’s *t*-test. Correlation by the Pearson test was used for univariate analysis between some cardiovascular risk factors and DMF%. Multivariate analysis was performed with linear regression between cardiovascular factors and %FMD, when univariate analysis showed statistical differences. The average and standard error of the mean (SEM) were respectively used as central tendency and dispersion measures.

## 3. Results

### 3.1. Demographic and Basal Characteristics

Data are shown in Table 1. Lithiasis patients and controls were age and sex matched. The average age was 52.1 years old, being two-thirds men, and one-third women. The BMI average was 26.82 Kg/cm^2^. Both groups were homogenous in anthropometric and exploration measures (MAP). In total, 12 (22%) patients were smokers, 8 in the patients’ group, and 4 in the control group. Stone characteristics from the medical record are provided in Table 2, being 15 (55%) patients relapsed and 19 (70%) with multiple stones. In 26 (96%) patients the stones were located in the kidney, being in 23 of them (85%) composed of calcium. 

No differences were found between both groups in all laboratory measures analyzed (glucose, triglycerides, total cholesterol, and HDL and LDL cholesterol). The Framingham risk was low in both groups, without differences.

### 3.2. Endothelial Function

**Figure 1:**
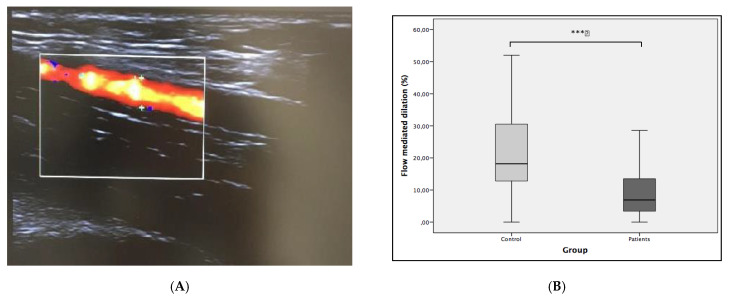
(**A**) Eco doppler capture of brachial artery. (**B**) Comparative analysis of flow-mediated dilation (FMD%) between groups. Statistical differences were calculated with t test. *** *p* = 0.0001.

The percent FMD increase was 11.85% (SE: 2.78) lower in lithiasis group reaching significant statistical differences (*p* < 0.001). The basal lumen diameter was significantly lower in the control group, although post compression lumen was similar in both groups. Data are shown in Table 3 and Figure 1.

For the study of the influence of the other demographic and analytic features, a univariate analysis was performed. A Student’s *t*-test was used for binary variables, whereas a correlation test was used for quantitative variables. The analysis also revealed that smokers showed a lower FMD, with a difference of 9.07% (SE: 2.62), without any differences in the rest of variables (age, gender, BMI, MAP, glucose, total cholesterol, and atherogenic index). Data are shown in Table 4. The stone characteristics did not show a relationship either with FMD% (Table 5).

The Student’s *t*-test FMD comparison between groups, excluding smokers, also revealed a statistically significant decrease of 12.3% (SE: 3.30, *p* = 0.001). Multivariate analysis, performed with linear regression, introducing smoking and the group (urolithiasic or control) as independent variables, and FMD as a dependent factor, also showed statistical differences between groups in both the variables of smoking (6.75%, SE: 3.3, *p* = 0.046) and lithiasis, which showed a 10.85% FMD decrease (SE: 2.74, *p* < 0.001). These results confirmed the independent significant statistical influence of urolithiasis and smoking in a lower FMD.

### 3.3. Inflammation, Oxidative Stress, and Endothelial Dysfunction Markers

Different markers implied in the oxidative stress-mediated inflammatory injury, and associated with the endothelial dysfunction, were selected for the recognition of serum markers. CRP and IL-6 were selected as inflammatory markers [10], MDA, also known as thiobarbituric acid-reactive substances (TBARS), as a recognized oxidative stress marker [11], VCAM-1 as an adhesion molecule and inflammatory marker used in the coronary artery injury associated with endothelial dysfunction [10], and ADMA as an endogenous competitive inhibitor of nitric oxide synthase linked to endothelial dysfunction and atherosclerosis [12].

As shown in Table 6, none of the serum markers showed statistical differences between groups. Nevertheless, CRP, VCAM, and ADMA showed an increase in lithiasic patients without reaching statistical differences.

**Figure 2:**
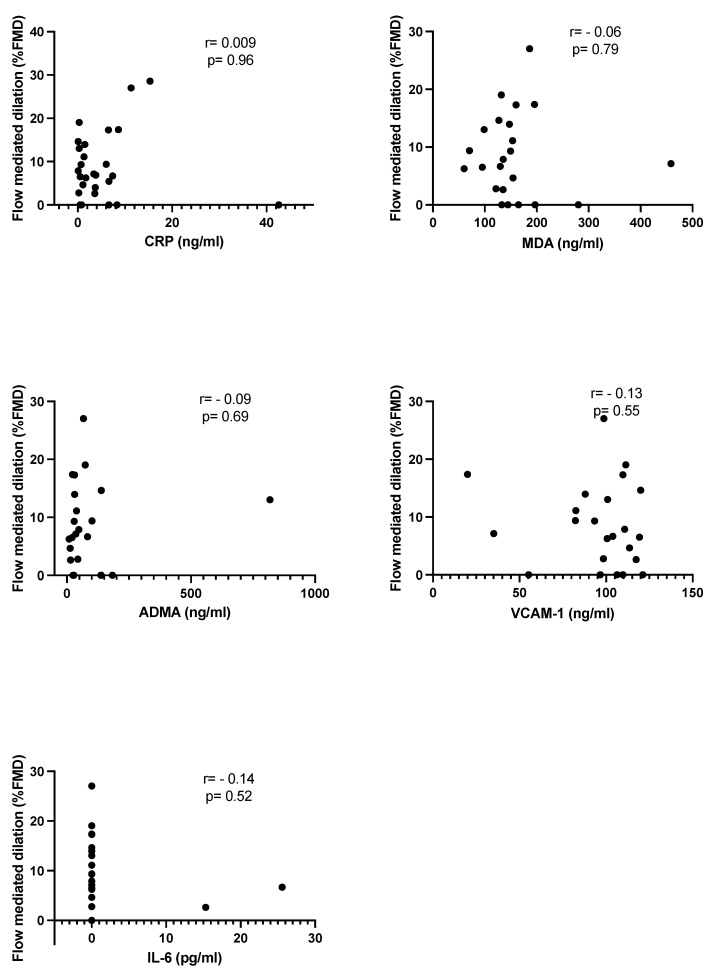
Group of study (lithiasic patients). Linear correlation between serum markers and DMF%.

The correlation analysis for the study of the behavior of the different molecules in relation with FMD in lithiasic patients, did not show statistical significance in any of them, although a negative correlation trend was observed in IL-6, MDA, and VCAM-1 (Figure 2).

## 4. Discussion

Endothelial dysfunction (ED) has recently been reported as a clinical feature in urolithiasic pathology in clinical studies [13,14]. Our data demonstrate that FMD is clearly impaired in lithiasic patients, persisting after controlling for confounding factors.

ED is a well-established disorder considered a key element in the development of atherosclerosis cardiovascular disease. On the other hand, a close relationship has been reported between the proximal tubular renal epithelium and the vascular endothelium with respect to the ion transport [15], which could be responsible for the relationship between urolithiasis and ED.

It was reported, in a large longitudinal study with 19,678 patients with a history of kidney stones, that there is a higher cardiovascular risk among women, with a multivariable hazard ratio of 1.18 (95% CI, 1.08–1.28) [2].

As shown in Table 3, FMD was 11.85% lower in lithiasic patients than in the control group. Interestingly, basal radial artery lumen has also demonstrated differences, being slightly higher in the lithiasic group. This circumstance can also be explained by the abnormalities in vascular reactivity and the lower response to the vasoactive factors of ED-affected arteries, seen in other pathologies such as diabetes [16,17].

A univariate analysis of possible confounding factors revealed that smoking is also a factor correlated to ED, as has been widely reported elsewhere, even in young patients without other confounding factors [18]. Both the linear regression and comparison between groups, excluding smokers, reported that FMD was significantly lower in the lithiasic group and, therefore, lithiasic pathology must be considered as an independent factor associated with ED. These results are in line with those reported by Yencilek and Yazici [13,14], in which they demonstrated the association between urolithiasis and a significantly lower FMD.

Subclinical inflammation and augmented reactive oxygen species (ROS) production has consistently been shown in ED. Some proinflammatory cytokines, such as IL-6 or CRP, have been also implied in the impairment of the endothelial function [4]. ADMA, as an endogenous nitric oxide synthase inhibitor, MDA, as a marker of OS through the lipid peroxidation pathway, and VCAM-1, as a protein implied in the inflammatory process of the vascular endothelium, were all evaluated as serum markers in the present study. The comparison of the values between both groups did not show any difference in any of the markers evaluated. None were associated with FMD, when correlations were performed in the lithiasic group (Figure 1), so none of these ED-pathogenic markers should be considered as serum predictors of FMD decrease in urolithiasic patients.

Previous studies have reported elevated levels of IL-6 and CRP in pathologies with low-grade inflammatory status, such as proteinuric diabetic nephropathy [19], or atherosclerosis and cardiovascular morbidity [20]. On the other hand, it has been reported that inflammation in renal injury is a factor that significantly contributes to stone formation [21]. In our study, no differences in CRP or IL-6 values were achieved between both groups. To the best of our knowledge, no studies have been performed with a direct comparison of absolute values between renal stone patients and controls.

Detectable inflammatory serum markers have been reported in renal stone patients [10], and in diabetic patients with urolithiasis compared to diabetic patients without urolithiasis [22]; the latter supports our recent findings that metabolic disease exacerbates local kidney inflammatory reaction in a rat model of hyperoxaluria [23]. Other studies, however, did not find any significant differences in serum values of inflammatory markers amongst four groups of patients: control, metabolic syndrome, urolithiasis, or both [24], similar to the present study. On the other hand, the absence of correlation between CRP and FMD in lithiasis patients initially rules out systemic inflammation as a pathogenic step causing endothelial dysfunction in renal stone patients, and therefore, does not allow us to use it as an inflammatory serum marker of endothelial dysfunction in urolithiasis.

It has been reported that renal epithelial injury may be a result of the influence of OS on the kidneys of idiopathic renal stone patients [25]. Moreover, our group recently reported higher levels of superoxide, mostly derived from NOX-1 in the kidney cortex and renal interlobar artery from an ethylene glycol-induced rat model of urolithiasis [26]. MDA is one of the most commonly used biomarkers for lipid peroxidation and oxidative stress. OS serum markers have been studied in patients with DM [11], hypertension, dyslipidemia, or obesity, but no studies have assessed serum MDA levels in patients with renal stones. In our study, no differences were found between groups, nor did we find a correlation with FMD. OS damage was initially considered as a local kidney phenomenon, and has been usually quantified in urine through markers such as 8-isoPGF_2_α [11]. Future studies are needed to elucidate the relationship between the local process of kidney stone formation and ED as a systemic effect.

A possible role of vascular endothelium on stone formation through NOS on epithelium ion transport has been suggested previously [15]. Aydin [5] firstly showed the effect of endothelium on calcium oxalate (CaOx) stone formation and outlined an increased local and systemic ADMA in an ethylene glycol-induced hyperoxaluria rat model. ADMA, as an endogenous competitive inhibitor of NOS, has been linked to ED, atherosclerosis, and mortality in patients with peripheral arterial disease [27]. In our study, no significant differences in ADMA serum levels were found between the study groups, although there was a trend for higher values in lithiasic patients. These findings are consistent with those reported by Yacizi in a similar clinical study with 76 patients [24]. As ADMA has been related to mortality in patients with advanced arterial disease, our patients were probably in an earlier stage, and FMD was thus a better predictor of ED in our patients. Nevertheless, studies with larger cohorts of patients must be performed in order to characterize the role of ADMA as an ED predictor of lithiasic patients.

The implication of macrophage populations in the calcium oxalate crystal formation has been reported in ex vivo models, and the facilitation of crystal formation by M1 macrophages and the protective role of M2 has been described [28]. On the other hand, our group reported that both MCP-1 and NFkB1 enhanced expression correlated to changes in oxidative stress levels and Nox1 upregulation in an ethylene glycol-induced rat model of urolithiasis [26]. VCAM-1 is considered as a biomarker and mediator in several cardiovascular disorders, including hypertension, stroke, and coronary heart disease, and is increased in ED [29]. It is considered to be the best marker for early atherosclerosis as an adhesion molecule [30]. It is a transmembrane molecule that mediates the immune cells to the vascular endothelium and can promote monocyte chemotaxis [31]. In our study, no significant differences were found despite a trend for higher levels in the urolithiasis group; in the same way, it was not possible to establish a significant correlation with FMD, although a slight inverse correlation has been detected. Tsao [10] reported a 36% sensitivity of detecting acute inflammatory response in a single arm study with 33 lithiasic patients. Thus, VCAM-1 is not probably useful as an isolated serum marker of ED in these patients.

FMD has been revealed as an important diagnostic tool for detecting ED in urolithiasic patients. Our findings, along with those reported by Yazici [14] and Yencilek [13], unequivocally show a lower FMD in patients with kidney stones. As this study was performed as a transversal design, it as not possible to determine whether urolithiasis is a consequence of a systemic low-grade inflammation status with enhanced stress, leading to ED, or in contrast, if urolithiasis must be considered as the initial disorder that triggers systemic effects, such as ED.

## 5. Conclusions

We can conclude that ED constitutes an important disorder in urolithiasis patients that should be regarded as a sentinel symptom of a cardiovascular morbidity, and a strong predictor of future cardiovascular events.

## Figures and Tables

**Table 1:** Demographic and laboratory measurements of study population (mean ± SEM).

	Patients	Controls	*p*
Age (years)	51.92 ± 2.91	52.33 ± 4.03	0.9350
Gender (F/M)	18/9	18/9	
Height (cm)	169.67 ± 1.86	167.15 ± 2.42	0.4141
Weight (kg)	77.28 ± 2.49	73.7 ± 2.74	0.3375
BMI (kg/m^2^)	26.88 ± 0.85	26.76 ± 4.79	0.9325
Smoking	8/27	4/27	0.526
TAM (mmHg)	109.68 ± 3.4	101.69 ± 3.04	0.0854
Glucose (mg/dL)	90.46 ± 3.26	93.63 ± 3.21	0.4251
Total Cholesterol (mg/dL)	188.48 ± 8.3	178.96 ± 5.93	0.3553
Triglycerides (mg/dL)	114.8 ± 14.26	92.04 ± 8.1	0.1807
HDL Cholesterol (mg/dL)	53.77 ± 3.05	59.81 ± 2.62	0.0970
LDL Cholesterol (mg/dL)	100.7 ± 7.11	99.5 ± 5.65	0.1592
Atherogenic Index	0.28 ± 0.07	0.16 ± 0.04	0.075
Framingham Score (%)	6.4 ± 1.52	7.45 ± 1.67	0.64

**Table 2:** Stone characteristics.

	*n*
Disease stage (First episode/Relapsing)	12/15
Multiplicity (Single stone/Multiple)	8/19
Location (Kidney/Ureter)	26/1
Composition	
Calcium	23
Uric acid	2
Unknown	2

**Table 3:** Endothelial function features (mean ± SEM).

	Patients	Control	*p*
Basal lumen diameter (mm)	4.33 ± 0.15	3.94 ± 0.11	0.0491
Lumen diameter after FMD (mm)	4.71 ± 0.17	4.73 ± 0.12	0.9301
FMD (% increase)	8.95 ± 1.51	20.81 ± 2.34	0.00010448

**Table 4:** Univariate analysis between demographic features and FMD% (Student’s *t*-test or correlation test).

	R (Pearson)	Mean Difference (T Test)	*p*
Age	−0.12706051		0.36
Gender		0.34	0.92
BMI (kg/m^2^)	0.07183757		0.6
Mean arterial pressure (mmHg)	−0.07209692		0.6
Smoking		−9.07	0.001
Glucose (mg/dL)	0.10709456		0.44
Total cholesterol (mg/dL)	−0.05018417		0.72
Atherogenic index	−0.06		0.64
Framingham score (%)	0.043		0.76

**Table 5:** Univariate analysis between stone characteristics and (FMD%) (Student’s *t*-test and ANOVA test).

	*n*	FMD (%)	*p*
Disease stage (First episode/Relapsing)	12/15	7.5/10.1	0.405
Multiplicity (Single stone/Multiple)	8/19	10.6/8.3	0.5
Location (Kidney/Ureter)	26/1	9/6.9	0.8
Composition			
Calcium	23	9.4	0.8
Uric acid	2	6.5	
Unknown	2	6.6	

**Table 6:** Inflammation, oxidative stress, and endothelial dysfunction markers (mean ± SEM).

	Patients	Control	*p*
CRP (mg/L)	5.3 ± 1.6	3 ± 0.9	0.2432
IL-6 (pg/mL)	1.7 ± 1.2	18.8 ± 13.8	0.23
MDA (ng/mL)	157.8 ± 15.3	199.57 ± 29.6	0.23
VCAM-1 (ng/mL)	95.4 ± 5.1	90.1 ± 4.8	0.47
ADMA (ng/mL)	92.8 ± 31.8	75.6 ± 30.2	0.71

## Data Availability

The datasets generated and/or analyzed during the current study are available from the corresponding author on reasonable request.
